# Measuring representation in clinical trials: Simulations demonstrating how current methods fail in the context of precision medicine

**DOI:** 10.1371/journal.pone.0342711

**Published:** 2026-03-10

**Authors:** Andrew Friedson, Abigail Humphreys, June Cha

**Affiliations:** 1 Milken Institute, Washington, District of Columbia, United States of America; 2 Milken Institute, New York, New York, United States of America; University of Health and Allied Sciences, GHANA

## Abstract

Clinical trial representativeness is vital for the evaluation of intervention performance and study generalizability. Current methods for evaluating representativeness are limited by the quality of the disease registry data used and may not appropriately evaluate studies aimed at precision cohorts. This study evaluates the sensitivity of existing methods for measuring study representation to differences between the population targeted by the clinical intervention and the closest available population in a patient registry. Using records for U.S.-based cancer clinical trials registered to ClinicalTrials.gov from 2017–2023 and the U.S. Cancer Statistics Public Use Database we calculated representativeness measures by comparing the demographic mix (based on sex, race, and ethnicity) in each clinical trial to the demographic mix for the same form of cancer in the U.S. Cancer Statistics database via exact Binomial tests. Then, the same tests were conducted comparing trial populations to simulated populations that were demographically different from the U.S. Cancer Statistics data by fixed percentages. The outcome of interest was whether the result of the test changed when the comparator population was different. For clinical trials reporting the sex, race, and ethnicity of participants, 24, 40, and 32 percent of studies (respectively) give different results when the difference between the registry population and simulated population is 5 percentage points. For all demographics, larger differences between the registry and the simulated populations were associated with worse metric performance. Analyses of clinical trial representativeness suffer from a large loss of accuracy in settings where treatments are targeted to demographically different subgroups. Studies of representation in the context of precision medicine should be interpreted with caution.

## Introduction

Clinical trials aim to evaluate the safety and effectiveness of new medical products—such as drugs, biologics, and medical devices—by examining their impact on human health. A trial’s ability to determine the clinical attributes of a new product depends on the credibility of the experimental design and the strength of the findings. These findings are only generalizable (i.e., externally valid) to populations that match the characteristics of the trial participants.

It is thus important for clinical trials to have appropriate representation, certainly in terms of the biological features of the trial participants but also ideally in terms of the social and environmental factors that influence both participant health and how the medical products under study are used, ultimately affecting their safety and efficacy [1]. Appropriate representation minimizes the difference between product performance in a trial and eventual performance when deployed in the field.

There have been many cases where the effectiveness of a medical intervention was found in post-market clinical use to have reduced effectiveness for specific demographics. To illustrate, consider two interventions aimed at cardiovascular illnesses that have reduced effectiveness in subpopulations that were discovered during post-approval use: Clopidogrel, which has been shown to be less effective for individuals with CYP2C19 loss-of-function alleles (which are more common in individuals of African and East Asian ancestry), and Angiotensin-Converting Enzyme inhibitors, which have been shown to be less effective in Black populations [2–6].

This raises a practical question: “How does one measure whether or not a clinical trial is representative?” On its surface, this is a straightforward statistical exercise. Assume that the characteristics of the clinical target population (the population with the health issue that the intervention under study is seeking to treat) are known. In that case, an exact Binomial (or related) test can be used to determine if a group of study participants is statistically different from the target population. This logic underpins a sizable and growing literature on assessing the representativeness of clinical research. This literature is largely, but not exclusively, focused on cancer and relies on data from patient registries to provide information on the clinical target population [7–20].

This research strategy has an important shortcoming. If the clinical target population used as the comparator is incorrect, then any subsequent classification of studies into “appropriately representative” or “not appropriately representative” may also be incorrect. This is precisely the problem facing the literature on representation in cancer studies: representation analyses compare study participants to the population who have the same form of cancer in a cancer registry. However, in recent oncology product development, novel medical products have been increasingly specialized to address genetically diverse mutations in cancer cells, even within the same cancer type [21]. These specialized therapies can offer more precise treatment with fewer side effects. Therefore, the clinical trials for specialized oncology product development are not necessarily aimed at the entire disease population (as is used as the comparator in evaluations of representativeness) but rather at a subpopulation for which the intervention is specifically tailored.

These subpopulations can have demographics that are different than the broader population with the disease. An example is triple-negative breast cancer (TNBC), where cancer cells do not have estrogen or progesterone receptors and make little or no HER2 protein. Based on the California Cancer Registry, the population with TNBC has 4.4 percentage points more Black individuals (and 4.1 percentage points more Hispanic individuals) than the population with any form of breast cancer [22]. When the demographic composition of a clinical trial’s target population (such as a study of TNBC) significantly differs from that of the broader disease population (such as breast cancer in general), commonly used representativeness metrics that compare the study population to the broader disease population will not accurately reflect the trial's generalizability. TNBC is not the only such example, de novo metastatic hormone sensitive prostate cancer has a much greater proportion (10.7 percentage points) of Black patients than the general prostate cancer population, and metastatic colorectal cancer cases have a greater proportion of male patients (7.9 percentage points) than the general colorectal cancer population [23,24].

This study explores the sensitivity of standard measures of representativeness when the target population differs from the population that is found in a patient registry. In other words, we ask the question, “What if the trial was aimed at a group with a genetic mutation that is on average X percentage points different demographically from the general disease population?” As X is increased, the number studies that would be incorrectly classified with existing methods is tracked.

We find that standard assessments of representativeness are highly sensitive to this type of error. The rate of misclassification grows rapidly as the gap between the clinical trial’s target population and the utilized registry population (which we refer to as the “relevant clinical population difference”) widens. These findings show that as novel medical interventions are increasingly specialized and focus on narrower patient subgroups, there is a parallel need for specialized comparators for assessing their representativeness.

### Study data and methods

#### Data sources.

Our first source of data is the Clinical Trials Transformation Initiative’s Aggregate Analysis of Clinical Trials (AACT) database [25]. The AACT is a machine-readable snapshot of all data posted to ClinicalTrials.gov. From the AACT we collected information from all cancer-related clinical trials that were listed as starting between January 1, 2017 and December 31, 2023. As of January 2017, the Food and Drug Administration (FDA) required all pharmaceuticals seeking approval to post their clinical trials and eventual results on ClinicalTrials.gov as part of the Final Rule of the Food and Drug Administration Amendments Act of 2007. From January 2017 onwards, the postings to ClinicalTrials.gov can be seen as a census of trials for drugs planned to go to market (contingent on successful trials and subsequent FDA approval).

**Fig 1:**
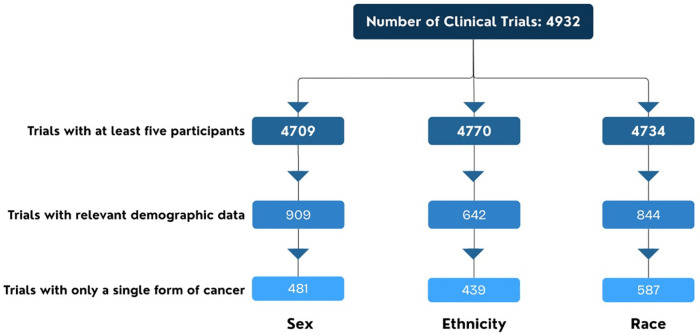
Sample selection process.

We then made three additional sample restrictions. We first restricted our sample to clinical trials with at least five participants. We then restricted to trials that had reported their results and that the results contained relevant demographic information. Finally, we restricted our sample to clinical trials that reported a single form of cancer so that we could accurately match to a single population in the cancer registry data. This left final samples of 481 trials reporting results that contained information on participant sex, 439 trials reporting results that contained information on participant ethnicity, and 587 trials reporting results that contained information on participant race. Fig 1 illustrates the sample selection process.

Our second data source is the U.S. Cancer Statistics Public Use Database, which covers cancer cases diagnosed between January 1, 2001, and December 31, 2021, in its most recent release [26]. We limit our sample to cancer cases diagnosed between 2017 and 2021. The database contains de-identified cancer incidence data reported to the Centers for Disease Control and Prevention’s National Program of Cancer Registries and the National Cancer Institute’s Surveillance, Epidemiology, and End Results Program by hospitals, physicians, and laboratories. These data report over 37 million cases across all 50 states and the District of Columbia. For each case, we can observe information on the type of cancer, as well as the patient sex, race, and ethnicity.

All the data used are publicly available and aggregated in a manner that no personally identifiable information is recoverable. As such, this study is not human subjects research.

#### Key variables.

For each clinical trial in our AACT sample, we are interested in the number of trial participants, *N*, as well as how many of those participants are female, how many are Hispanic, and how many are Black (these sex, ethnicity, and race categories are not mutually exclusive, but intersections of the categories are not consistently reported in the data). This allows us for any given demographic to say that a clinical trial had *k* participants of a given demographic out of *N* total. For each clinical trial, we also observe the type of cancer that the medical intervention was attempting to treat.

From U.S. Cancer Statistics, for each type of cancer that we can match to a clinical trial, we collect the total number of people reported with that form of cancer as well as the number of people in each demographic who are part of that cancer population. This allows us to calculate demographic ratios for each form of cancer. Table 1 lists the cancers we study, and the percent of the U.S. patient population captured in the U.S. Cancer Database that is female, Hispanic, or Black for each form of cancer.

**Table 1:** Demographics of U.S. cancer population.

Form of Cancer	Cases in U.S. Cancer Database	Female	Hispanic	Black
Brain and other nervous system	112,492	43.78%	11.44%	7.49%
Colon and Rectum	692,898	47.08%	10.44%	12.31%
Esophagus	89,819	20.95%	5.66%	7.96%
Hodgkin Lymphoma	40,118	45.06%	14.37%	13.25%
Kaposi Sarcoma	1,852	11.29%	41.36%	16.74%
Kidney and Renal Pelvis	333,318	36.29%	12.04%	12.08%
Larynx	56,765	20.79%	6.27%	14.04%
Leukemias	260,525	41.60%	10.53%	8.43%
Liver and Intrahepatic Bile Duct	176,562	29.96%	16.53%	12.89%
Lung and Bronchus	1,077,616	49.26%	4.99%	10.67%
Skin Melanoma	411,587	40.79%	2.49%	0.39%
Mesothelioma	13,381	26.93%	7.10%	2.38%
Myeloma	138,638	44.36%	9.91%	21.33%
Non-Hodgkin Lymphoma	352,906	44.59%	10.71%	8.13%
Oral Cavity and Pharynx	235,171	28.51%	6.84%	7.82%
Pancreas	268,317	48.00%	9.03%	12.57%
Thyroid	216,386	72.93%	16.01%	7.38%
Urinary Bladder	368,738	23.65%	5.27%	6.08%
Cervix	62,187	–	19.78%	14.88%
Female Breast	1,281,423	–	9.61%	11.75%
Female Breast, in situ	282,879	–	9.79%	13.28%
Corpus and Uterus, NOS	289,129	–	11.30%	12.71%
Ovary	99,084	–	12.12%	10.04%
Prostate	1,094,137	–	7.66%	16.37%
Testis	43,261	–	21.84%	3.26%
All Cancers listed Above	7,999,189	40.88%*	8.75%	11.11%

Source: U.S. Cancer Statistics Public Use Database

*Female percentage does not include single-sex cancers.

#### Overview of analysis.

Our analysis involves two steps. First, we measure the representativeness of each clinical trial according to established methods. To do this, for each clinical trial in our sample, we calculate the likelihood that the demographic proportion of interest in the study cohort could arise from random draws from the corresponding disease population observed in the cancer registry. We then mark studies with a less than 5 percent chance of being a random draw as “not likely representative.” Details on executing this statistical exercise are provided in S1 Appendix, but in general, the exercise is analogous to testing via exact binomial confidence intervals as is done in the literature when evaluating individual studies [7].

The second step is to ask the question, “How would the outcome of the exercise change if the target population for the clinical trial was different from the disease population found in the cancer registry?” To answer this question, we repeat the analysis above but instead of comparing the clinical trial demographic balance to the relevant population in the U.S. Cancer Database, we use a value that is X percentage points different from the U.S. Cancer Database value, with X ranging from −15–15 in increments of 1. This can be seen as a simulated disease sub-population with a known demographic difference from the general disease population. Each iteration of this exercise asks, “Which studies would be considered not likely representative if the relevant clinical population (the simulated disease sub-population) was X percentage points different than the general disease population reported in the U.S. Cancer Database?” We refer to this X as the relevant clinical population difference: the percentage point difference between the demographic mix in the disease subpopulation of interest, and the demographic mix in the larger disease population.

For each relevant clinical population difference, we count the proportion of clinical trials that switched from “not likely representative” to “likely representative” or vice versa. This tells us that if the relevant clinical population is X percentage points different than the population in the U.S. Cancer Database, then what proportion of studies would a standard method for gauging representativeness misclassify? When we refer to a study as misclassified, we mean either that the study was representative and standard measures would mark it as not representative or that the study was not representative and standard measures would mark it as representative.

When looking at single-sex, or close to single-sex cancers (cancers of the cervix, uterus, ovary, prostate and testis), we conduct the analyses for ethnicity and race but not sex. All analyses were conducted using StataSE 18 software.

## Results

**Fig 2:**
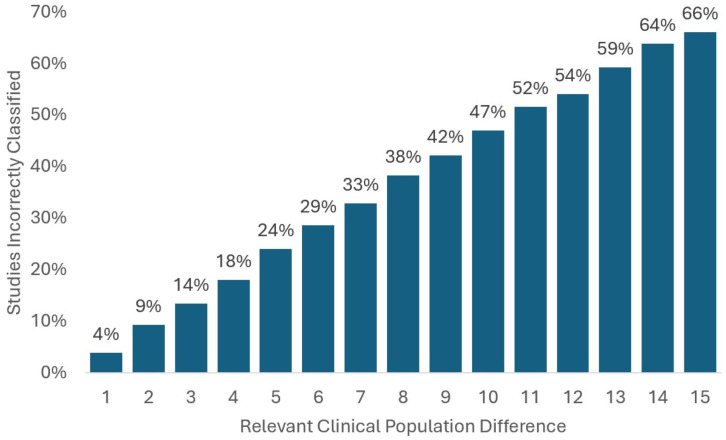
Misclassification of study representativeness based on sex (of 481 studies). Source: Authors’ analysis of data from the AACT Database and U.S. Cancer Statistics Public Use Database.

Misclassification based on sex is frequent, even at small relevant clinical population distance. When there is a 5-percentage point difference between the clinical target population and the percentage of females in the relevant cancer category according to the U.S. Cancer Database, just under a quarter of studies would be misclassified. Results for sex for all simulated relevant clinical population differences are shown in Fig 2.

The number of misclassifications steadily increases as the relevant clinical population difference increases. At a relevant clinical population difference of 15 percentage points, two-thirds of studies are misclassified. For example, if we tried to gauge clinical trials targeted to metastatic colorectal cancer cases (which are 39.2 percent female) based on general colorectal cancer case demographics (which are 47.1 percent female, yielding a relevant clinical population difference of 7.9 percentage points), we would incorrectly classify over a third of studies [24].

**Fig 3:**
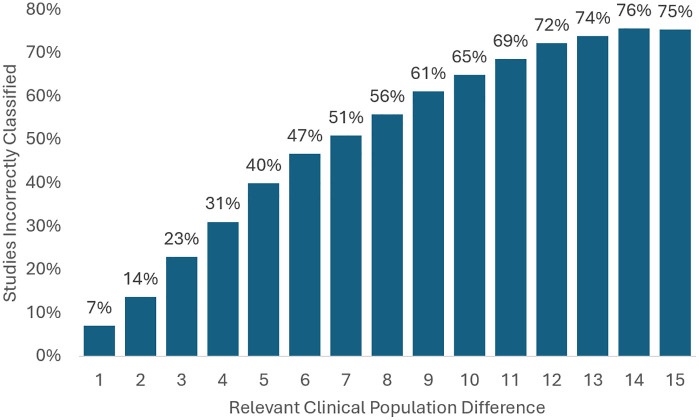
Misclassification of study representativeness based on ethnicity (of 439 Studies). Source: Authors’ analysis of data from the AACT Database and U.S. Cancer Statistics Public Use Database.

The number of misclassified studies increases more rapidly with relevant clinical population difference for ethnicity than it did for sex (Fig 3). At a relevant clinical population difference of 5 percentage points, a quarter of studies were misclassified based on sex. Based on ethnicity, at a relevant clinical population difference of 5 percentage points, 40 percent of studies are misclassified. At a relevant clinical population difference of 15 percentage points, three-quarters of studies are misclassified.

**Fig 4:**
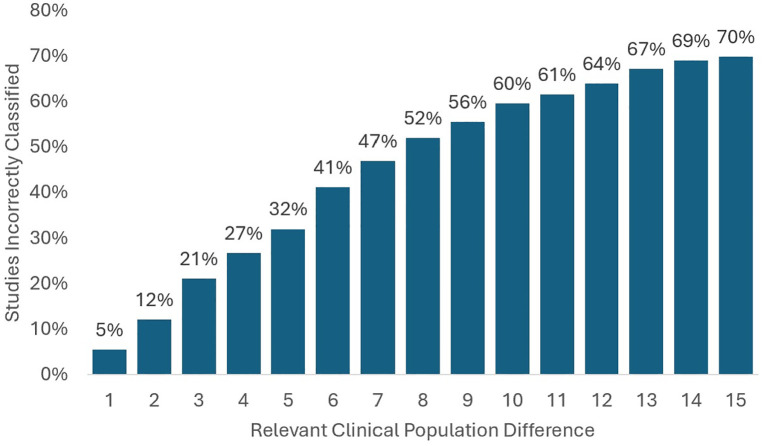
Misclassification of study representativeness based on race (of 587 studies). Source: Authors’ analysis of data from the AACT Database and U.S. Cancer Statistics Public Use Database.

The proportion of misclassified studies grows faster for race than for sex, but slower than for ethnicity. At a relevant clinical population difference of 5 percentage points, just under a third of studies are misclassified based on the proportion of the study that is Black. At a relevant clinical population difference of 15 percentage points, 70 percent of studies are misclassified (Fig 4). We also repeat the analysis for the percent of a population that is Asian, which can be found in S2 Appendix.

Overall, a small difference in the demographic makeup of the target population relative to the demographics found in a registry can result in misclassification of a large proportion of studies using standard measures. For example, if we tried to gauge clinical trials targeted to TNBC cases based on general breast cancer racial demographics we would incorrectly classify over a quarter of studies [22]. The misclassification problem can be worse, a more extreme case is de novo metastatic hormone sensitive prostate cancer, which has a much greater proportion of Black patients than the general prostate cancer population (a relevant clinical population difference of 10.7 percentage points) [23]. Using the broader prostate population in this case would misclassify around 60 percent of studies.

## Discussion

This study demonstrates that the results of studies that measure the representativeness of clinical research should be interpreted with caution, especially in situations where the clinical research in question looks at interventions that are targeted at demographically differentiated subpopulations. We provide important nuance to the discussion of representation in research especially in the context of the push toward more individualized product development.

As more patient-focused strategies aimed at tailoring treatments for people with specific biomarkers come online, precision medicine will continue to diverge from the historical ‘average patient’ model by drawing on each patient’s genetic profile, environmental context, and lifestyle factors [27].This precision approach enhances treatment accuracy and illuminates the biological mechanisms behind disparities in disease outcomes, but also makes standard measures of representativeness more likely to draw incorrect inference. Our results are novel in that they quantify this measurement shortcoming directly.

There are several limitations to our study. The first is that the above analysis is an evaluation of a single type of representation measure for a single disease. These results are generalizable only in contexts where similar demographic patterns exist, namely for diseases where there are clinically relevant sub-groups that are demographically different from the broader disease population. To the extent that this is true, this exercise would be likely to be replicable in those contexts.

There are also limitations based on the demographic measures available. We are limited to measures that are recorded to ClinicalTrials.gov as well as in U.S. Cancer Statistics Database. This means that more complete measures of individual demographics (which may be clinically important if tied to specific genotypes) cannot be used, and that the demographic measures used do not necessarily line up with those that would be found in other countries.

A fundamental question emerging from this exercise is, “How do we develop more robust yardsticks for trial representativeness that accurately capture the clinical trial target population?” Addressing this challenge calls for a combination of high-tech and low-tech approaches. On the high-tech side, data aggregation and artificial intelligence (AI) tools are transforming how researchers capture patient data from medical records and create cohorts that match the characteristics needed for precision medicine interventions [28]. Tools such as these allow researchers to find more reliable ways to identify the relevant clinical population and could serve as the standard for constructing representativeness metrics in precision medicine.

These data tools come with important caveats. Generating custom cohorts from medical records requires that the people who need medical care can both access healthcare and have their information accurately recorded in the first place. This reliance on recorded data inadvertently excludes those outside the healthcare system due to affordability or limited access. For the high-tech tools to be effective, they need to be paired with lower-tech, on-the-ground intervention via patient outreach, engagement, and community-based support to ensure that genomic datasets are themselves complete and representative of the relevant patient populations.

## Conclusion

Representativeness metrics are highly sensitive to even small discrepancies between the “true” clinical target population and the population used as a reference. Therefore, we would advise caution in using them to evaluate trials unless higher-precision population data is available. By integrating advanced data-driven methods with community-based outreach and engagement, we can ensure that measures of representativeness accurately reflect the various patient groups intended to benefit from precision medicine.

## Supporting information

S1 Appendix Statistical Techniques – A detailed explanation of the statistical techniques. (DOCX)

S2 Appendix Misclassification of Study Representativeness Based on Race (Asian). (DOCX)

S3 File Replication Package – Contains dataset and replication code. (ZIP)
