# Supplementary material for: Measuring representation in clinical trials: Simulations demonstrating how current methods fail in the context of precision medicine
Source: PLoS One. 2026 Mar 10;21(3):e0342711. doi: 10.1371/journal.pone.0342711 (PMC12974884; doi:10.1371/journal.pone.0342711)
Supplement: S1 Appendix — (DOCX) [file pone.0342711.s001.docx]

**S1 Appendix. Statistical Techniques**

The first step of our analyses is to assess if the observed demographic mix in a clinical trial with *N* participants is representative of the demographic mix in the underlying population of people with the disease being studied. The underlying population has some known demographic mix, where *q* such that $0\leq q\leq1$is the proportion of the underlying population that is a member of the demographic of interest. Then, for each person participating in the clinical trial we can ask the yes or no question “is this person a member of the demographic of interest?”

This makes the random selection of a person from the underlying population a *Bernoulli Trial* where a “success” is drawing someone of the demographic of interest and has the probability *q*. A random sample of size *N* would then be *N* independent Bernoulli Trials. Given this data generation process, the likelihood of sampling a clinical trial population of *N* participants where *k* of them are of the demographic of interest follows the *Binomial Distribution*. If the parameters *q*, *N,* and *k* are known (which in the instance of this analysis they are), the Binomial Distribution allows us to calculate the probability that given the underlying demographic prevalence of *q*, a trial of *N* participants has at least (or at most) *k* participants of the demographic of interest.

So, when a trial has a ratio *k/N* that is lower than *p*, we are interested in the probability that *N* draws had *k* or fewer members of the demographic of interest. Likewise, if a trial has a ratio *k/N* that is greater than *p*, we are interested in the probability that *N* draws had *k* or more members of the demographic of interest. In either of these cases, our null hypothesis is that the observed trial was randomly drawn from the underlying population (the study was classified as likely *representative of the underlying population*), and we can use the calculation from the Binomial Distribution to test the alternative hypothesis that the observed trial was not randomly drawn from the underlying population (the study was classified *not likely representative of the underlying population*). We can reject the null hypothesis and mark studies as not representative when the probability of the observed or a more extreme draw is less than the usual threshold for statistical significance or *p-value* of 0.05.

This is analogous to constructing an exact 95-percent Binomial confidence interval around the demographic mix in the study and checking to see if the demographic mix in the underlying population is inside of the confidence interval (in which case the study is *likely representative of the underlying population*) or outside of the confidence interval (in which case the study is *not likely representative of the underlying population*). This is the method used by Algrighetti et al. (2021), and we conduct this analysis for each trial in our data.

The second step is to iteratively repeat the above analysis but change the value of *p* by X percentage points*.* We increase and decrease *p* iteratively by 0.01 at a time up to a maximum of X = 0.15 and a minimum of X = -0.15. On each iteration, we record whether or not a study changes from the classification that was assigned at the original value of *p* (X = 0). Finally, we count up the proportion of studies that change classifications for each alteration of *p.* So, for an absolute value change of 0.3 (X = 0.03 or X = -0.03), we count up the proportion of studies that change classification when either 0.3 is added to or is subtracted from *p.*

References

Algrighetti CM, Niemierko A, Van Allen E, Willers H, Kamran SC. Racial and ethnic disparities among participants in precision oncology clinical studies. JAMA Network Open. 2021; 4(11): e2133205.
