# Supplementary material for: Measuring representation in clinical trials: Simulations demonstrating how current methods fail in the context of precision medicine
Source: PLoS One. 2026 Mar 10;21(3):e0342711. doi: 10.1371/journal.pone.0342711 (PMC12974884; doi:10.1371/journal.pone.0342711)
Supplement: S2 Appendix — (DOCX) [file pone.0342711.s002.docx]

**S2 Appendix. Misclassification of Study Representativeness Based on Race (Asian)**

Fig S2 shows the results of our analysis for racial representativeness when Asian is used as the demographic of interest. The x-axis shows the relevant clinical population difference (the percentage point difference between the percentage of Asian individuals in the clinical target population and the percentage of Asian individuals in the relevant cancer category according to the U.S. Cancer Database). The y-axis shows the share of studies posted to ClinicalTrials.gov that would be misclassified by standard representativeness measures. Using this method, when there is a 5-percentage point difference between the clinical target population and the percentage of Asian individuals in the relevant cancer category according to the U.S. Cancer Database, 45 percent of studies would be misclassified. This is the most rapid increase of any of the demographics tested in this study.

**Fig S2 – Misclassification of Study Representativeness Based on Race (Asian)**

Source: Authors’ analysis of data from the AACT Database and U.S. Cancer Statistics Public Use Database
